# Effects of neighborhood features on healthy aging in place: the composition and context of urban parks and traditional local coffeeshops in Singapore

**DOI:** 10.1186/s12877-022-03679-z

**Published:** 2022-12-15

**Authors:** Huso Yi, Shu Tian Ng, Cheng Mun Chang, Cheryl Xue Er Low, Chuen Seng Tan

**Affiliations:** Saw Swee Hock School of Public Health, National University of Singapore and National University Health System, Singapore, Singapore

**Keywords:** Built environment, Neighborhood effects, Older adults, Healthy aging in place, Exercise, Smoking, Ethnographic assessment, Mixed methods

## Abstract

**Background:**

Healthy aging in place is affected by what the neighborhood provides for older adults. The mixed-methods ethnographic study explored the built environmental and contextual effects of urban parks and traditional local coffeeshops (*kopitiam*) on health practices among older adults in Singapore.

**Methods:**

A door-to-door survey with 497 older adults from 32 residential blocks in a public housing town assessed exercise and smoking. The walking distances from the residential blocks to the facilities were calculated. Regression analysis examined the associations between the distance and rates of exercise and smoking. Ethnographic assessment data contextualized the quantitative findings.

**Results:**

Older adults’ exercise was associated with proximity to an urban park but not traditional local coffeeshops. High rates of smoking were clustered in the housing blocks close to the coffeeshops, which provided casual drinking places with smoking tables. The proximity to the coffeeshops was significantly associated with increased smoking and decreased exercise. A walking distance of 200 m to the park and coffeeshops was found to discriminate the outcomes.

**Conclusions:**

The findings suggested that walking distances of a few blocks influenced health behaviors among older adults. Their smoking habits appeared to be maintained through environmental features and cultural norms attached to the coffeeshops. Policy of urban planning and redevelopment for the aging population needs to consider the socioecology of healthy aging in place.

**Supplementary Information:**

The online version contains supplementary material available at 10.1186/s12877-022-03679-z.

## Introduction

Neighborhood built and social environments serve as a foundation of healthy aging in place – living in one’s own community with well-being, independence, and autonomy while maintaining meaningful connections to support systems with family, friends, and neighbors and access to community services and facilities [1]. The concept of healthy aging, albeit with no conceptual and empirical agreement, is commonly understood based on three dimensions of bio-social-psychological models [2–4]. In early research on the adaptation to aging, ‘successful aging’ was first created and conceptualized as the balance between the absence of disease and disease-related disability, high functional capacity, and active engagement with life [5]. Another frequently used term is active aging, defined as “continued participation in social, economic, cultural, spiritual and civic life as well as social, mental and physical well-being, autonomy and independence” [6].

For the past two decades, there have been efforts to define healthy aging operationally [4].The concepts of healthy aging are mainly based on the WHO definition of health as a ‘state of complete physical, mental and social well-being [2, 4]. For example, WHO defines healthy aging as “process of developing and maintaining the functional ability that enables well-being in old age” [7]. Pan American Health Organization, WHO defines health aging as “continuous process of optimizing opportunities to maintain and improve physical and mental health, independence, and quality of life throughout the life course” [8]. The European Healthy Ageing Project defines healthy aging as “process of optimizing opportunities for physical, social and mental health to enable older people to take an active part in society without discrimination and to enjoy an independent and good quality of life” [9].

In the operationalization of the concept, scientific efforts were made to develop validated scales of healthy aging. For example, the Aging Trajectories of Health-Longitudinal Opportunities and Synergies (ATHLOS) project constructed a 41-item healthy aging scale assessing intrinsic capacity and functional ability in biopsychosocial aspects of health to establish a standardized index across countries [10]. As the scale was initially developed to use in international epidemiological research settings, the scale was limited to assessing social and physical environmental aspects of healthy aging. With the advance of preventive and curative healthcare, the aging population is growing; there is increasing awareness of the importance of multidimensional social-psychological determinants of healthy aging, which include subjective meanings of and adaptation to aging, community and civic engagement, social and cultural capital, empowerment, and agency [4, 11–13]. Enabling ‘age-friendly’ environmental attributes plays an instrumental role in social well-being in healthy aging [14], including accessibility, proximity, and affordability [15]. The concept of healthy aging is useful in the discussion of urban planning policy for the aging population [14, 16].

With decreasing sphere of physical mobility and social relations, how older adults attach to the community is crucial for healthy longevity [17]. Their lives and identities are more physically constrained and neighborhood bound than younger adults, whose mobility and social ties are broader beyond neighborhood or kinship [18, 19]. Aging in place, if implemented effectively, delays functional and physical disabilities and improves psychological well-being and overall quality of life [20]. Consequently, it can reduce the cost of institutionalized care, which can then be allocated to promote community care [21, 22]. While it is favorably adopted in the policy of aging populations [23], research on the concept of aging in place is still warranted in terms of its constructs and assessments [24–26].

Accordingly, achieving the *desired* healthy aging in place for older adults is further complex than stated in its principle [27, 28]. If the policy of aging in place is not properly designed and implemented, it could rather increase frustration, social isolation, and negative emotions among older adults due to inappropriate adjustments at home and a lack of environmental design to remove physical restrictions and access community resources in the neighborhood [29–31], in particular among older adults with cognitive decline [32]. In particular, considerable challenges in the design of age-friendly communities have been addressed in terms of urban renewal for the aging population [33]. While modifying urban spaces can make existing neighborhoods more salubrious to older adults’ health, the extent to which interventions are feasible depends on the existing built conditions. While addressing policy and space constraints, older adults’ participatory engagement is crucial in the urban redevelopment of age-friendly environments [34].

In environmental gerontology, aging in place is constructed as a complex process with constant adaptations to all the changes in the body and sense of self, personal relations, neighborhood features (e.g., redevelopment and gentrification), and policies in the range from home to community [35–37]. Within the domestic domain, adequate age-friendly housing is necessary, enhancing their independence and preventing frailty by removing obstacles, adjusting amenities (e.g., kitchen and bathroom), installing mobility aids, and emergency responses [38]. Home maintenance and home-based care need to be accessible within the community [39, 40]. However, the provision of age-friendly housing is a minimum requirement for aging in place. In the immediate environment, older adults should be able to get around their neighborhoods and actively engage in daily activities with minimal assistance. Most importantly, besides community-based healthcare services, recreational, cultural, and educational resources and opportunities need to be provided [34]. How older adults feel emotionally about their neighborhood is central to their well-being in their later years.

As older adults spend most of their time in the community, they experience a greater sensitivity to neighborhood changes. Subjective feelings and experiences of adaptations are crucial, often regardless of objective measures of resources [41]. Social support systems, through the connectivity to neighborhood features, alleviate the negative impacts of changes (e.g., social exclusion) and build collective resilience with aging [42]. Thus, aging in place is made possible by fulfilling personhood supported by age-friendly infrastructure. The health of older adults is closely affected by what their neighborhood provides for them emotionally and how their community provisions are perceived and utilized by them [43, 44]. Social space for older adults is a result of the self-selected ongoing production of social relations. They prefer to select spaces and turn them into social or vernacular spaces through frequent use [45]. Attachment to such places encourages a sense of independence, belongingness, trust, support, and, therefore, agency for further positive changes. Proximity to community places is a key indicator as it facilitates active lifestyles physically and socially and creates social prescriptive norms of “what is considered to be healthy” among older adults [46].

The literature on neighborhood effects reflects the importance of accessibility to such facilities as older adults experience more time for recreational activities. Among these, parks have featured prominently, with studies reporting that living near a park increased the frequency of physical activity [47]. There is strong evidence of the benefits of physical activity in healthy aging, reducing the risk of developing chronic diseases, frailty, cognitive decline, depression and other mental illness, and mortality in older adult populations [48–50]. Parks provide exercise facilities, while the greenery also attracts residents who live nearby, creating opportunities for social interactions and collective health practices [51]. Walkability and connectivity are especially essential for older adults to engage in park-based physical activity [52, 53] and neighborhood social activities [54]. It is important to note that the relationship between accessibility and physical activity is confounded by neighborhood-level characteristics, such as neighborhood deprivation, social contexts, and perceptions of neighborhood, park quality, and street configurations [55, 56]. Thus, the contextualization of urban planning plays a crucial role in the effective utilization of parks and other greenery places for health promotion.

The harmful effects of smoking on healthy aging are evidenced, increasing the risk of cardiovascular and lung diseases, disability, and all-cause mortality in the population [57] and dementia and cognitive decline [58]. Despite the clinical benefits of smoking cessation among older adults [59], little is known about the social-ecological aspects of smoking among older adults. Among youths, proximity to tobacco outlets was associated with smoking as the outlets provided cues for smoking, access to cigarettes, or signal norms to encourage smoking [60, 61]. Smoking adults could attempt to preserve a neighborhood place for their own space in response to tobacco denormalization policy [62]. A study with older adults suggested smoking could be part of (mal)adaptative processes to cope with aging influenced by changes in surrounding environments; while they agreed with the benefits of non-smoking, they did not feel an attempt to quit smoking as “the damage was already done” [63]. A review suggested that older smokers were less likely to attempt quitting than younger smokers, but they were more likely to succeed in quitting if they decided to quit [64].

### Association between smoking and physical activity among older adults

Smoking and physical inactivity are the leading risk factors for morbidity and mortality in older adult populations [48–50, 57, 65]. With respect to the linkage between smoking and physical activity, it is anticipated that smokers decrease physical capacity and fatigue resistance for exercise; therefore, they are less likely to exercise regularly and be physically independent in later life [66, 67]. There is empirical evidence about the linkage between the two behaviors. A systematic review of the association between smoking and physical activity found that about 60% of reviewed studies reported a direct negative association between the two behaviors [68]. A 20-year prospective follow-up study found that the combined harmful effects of smoking and insufficient physical activity will increase the risk of frailty and disability, which in turn lead to death [69]. Across countries, population-based studies with large samples of older adults reported a negative association between smoking and physical activity in China [70], India [71], Poland [72], Sweden [73], and the US [74]. As smoking tends to be long-established among older adults as they initiate smoking at younger age, smoking status can predict physical activity [74]. The studies, however, noted that the degree of the associations differed by demographic characteristics (age, gender, education, and employment status), psychosocial status, and living environment.

### A study of urban parks and traditional local coffeeshops in Singapore

Singapore is one of the countries with the highest life expectancy. The country will soon become a “super-aged” society with a projection of 24% aged 65 years and above by 2030 [75]. In response, the government developed policies, such as “Action Plan for Successful Aging” and “Healthcare 2020 Masterplan”, to support healthy aging in place [76]. The Ministry of Health partners with the Housing and Development Board (HDB), which is responsible for public housing and looks for solutions in urban planning and the design of age-friendly towns. In 2007, “Neighborhood Renewal Program” was initiated in the public housing estates, including its goal to create age-friendly environments in line with the Enhancement for Active Seniors Program [77]. Community facilities that promote walkability, socialization, and mental stimulation among older adults are incorporated into existing and upcoming public housing estates.

Understanding the roles of neighborhood features in the context of HDB public housing is especially important in Singapore [78]. More than 80% of the population live in public housing, and 90% of public housing residents own their homes [77]. The public housing blocks are built to be self-contained towns, offering an urban infrastructure of commercial, recreational, and social amenities, and publicly subsidized community centers within walking distance. The features of public housing towns play an essential role in the well-being of older adults as they spend most of their time within their immediate neighborhood [79]. However, the effects of these urban renewal policies on aging in place are little known in terms of how older adults adapt to changing environments between newly introduced facilities and existing localities they have enjoyed for a long time [18].

In the current study, we chose two places in the public housing towns that are closely attached to older adults’ health and social life: modern urban parks and traditional local coffeeshops, known as *kopitiams*. Modern urban parks are designed to be integrated into the public housing towns for day-to-day recreation, physical activity, and social gathering, ‘fitting for a modern and progressive city state’ [80]. Concrete paths make the terrain conducive for strolling and jogging. Equipment like stretch bars and sit-up benches are provided for static exercises at fitness corners. There are also open spaces for exercise and community events, such as festival celebrations, organized by the public housing resident’s committees, which are state-affiliated organizations to promote social cohesion in the designated town.

The design of an urban park is especially important for the aging population in the country. Recently, to enhance holistic community care for aging in place with parks, the National Parks Board launched its first therapeutic gardens that recognized the rehabilitative benefits of horticultural therapy [81]. While the park’s characteristics are catered to visitors of all ages, the garden design and programs in the park are especially prioritized for healthy aging. For example, bench amenities include armrests for additional safety measures, and park features such as planting racks are designed at a height to allow easy accessibility to wheelchair users. Urban gardening and farming programs aim to promote social interaction and connection with nature for older adults.

The term “*kopitiam*” is a combination of the Malay term for coffee (*kopi*) and Hokkien term for a shop (*tiam*), which refers to a traditional coffeeshop in Singapore [82]. It is listed as an intangible cultural heritage by UNESCO. *Kopitiams* are smaller, privately-owned, open-air establishments with about 8 to 10 stalls selling food and beverages, alcohol, and cigarettes. Also referred to as eating houses or food courts, they are are mostly located on the ground floor of public housing blocks. Historically, the earliest forms of *kopitiams* were makeshift structures where itinerant hawkers sold affordable food and beverages to low-wage workers at plantations. With the arrival of Hainanese and Foochow communities in the early twentieth century, they began to be established in shophouses and grew in numbers as a respite where patrons enjoyed social bonding, political debate, and entertainment from radio and TV [83]. Following Singapore’s independence in 1965, the government developed massive public housing estates that provided *kopitiams* as an essential commercial facility in the town, located by public walkways. Despite the dominance of ethnically Chinese *kopitiams*, other ethnic food stalls, like Indian/Indian-Muslim food, and vegetarian options are always found. They are built to preserve the original identity of the affordability, inclusivity, and casual intimacy that older shop house *kopitiams* had, thereby appealing to older adults who possibly lived through that period. All the stalls share a communal seating area, allowing a group of patrons to have dishes from different stalls at one table.

Being a staple fixture in public housing towns, the crowd in a *kopitiam* represents a neighborhood’s makeup as diverse groups of people move in and out of place throughout the day. Most have televisions for entertainment and a designated smoking area, which is a notable attraction considering that the government has banned smoking in most public spaces [84]. The policy of tobacco demoralization makes it difficult for smokers to find a place to smoke, even at home, as the public housing units usually have screen doors for ventilation, and smoke spreads easily through the corridors [85]. Thus, *kopitiams* stand out by explicitly welcoming smokers by demarcating a designated smoking area space for them to be at ease.

### Study aims

Increasing geographical proximity and accessibility to social places that promote physical and mental well-being is a core issue in urban planning for an aging population. Due to limited mobility, older adults’ lives and identities are closely related to the composition and context of their neighborhood features. Overall, built environmental effects of the proximity to the park and tobacco retail place on physical activity and smoking are well-known. However, social-cultural understanding of these effects among older adults is less known, particularly in non-Western contexts. In Singapore, while the history and environmental settings of urban parks and traditional coffeeshops are substantially different, they both serve similar important social functions for aging in place. The study aims were to explore how the spatial location of public housing blocks in relation to parks and *kopitiams* affected the rates of exercise and smoking among older adults in our study neighborhood; and how the social-cultural context of the places affected their adaptation to health behaviors. The hypotheses tested in the quantitative phase were as follows: (1) physical proximity from the residence to the urban park would be associated with an increase of the frequency of exercise; and (2) physical proximity from the residence to kopitiam, which only provided designate smoking area in public gathering places, would be associated with an increase of habitual smoking. The research question explored in the ethnographic phase was how social-cultural aspects of place attachment and identity would affect exercise and smoking behaviors among older adults.

## Methods

### Study neighborhood

**Fig. 1:**
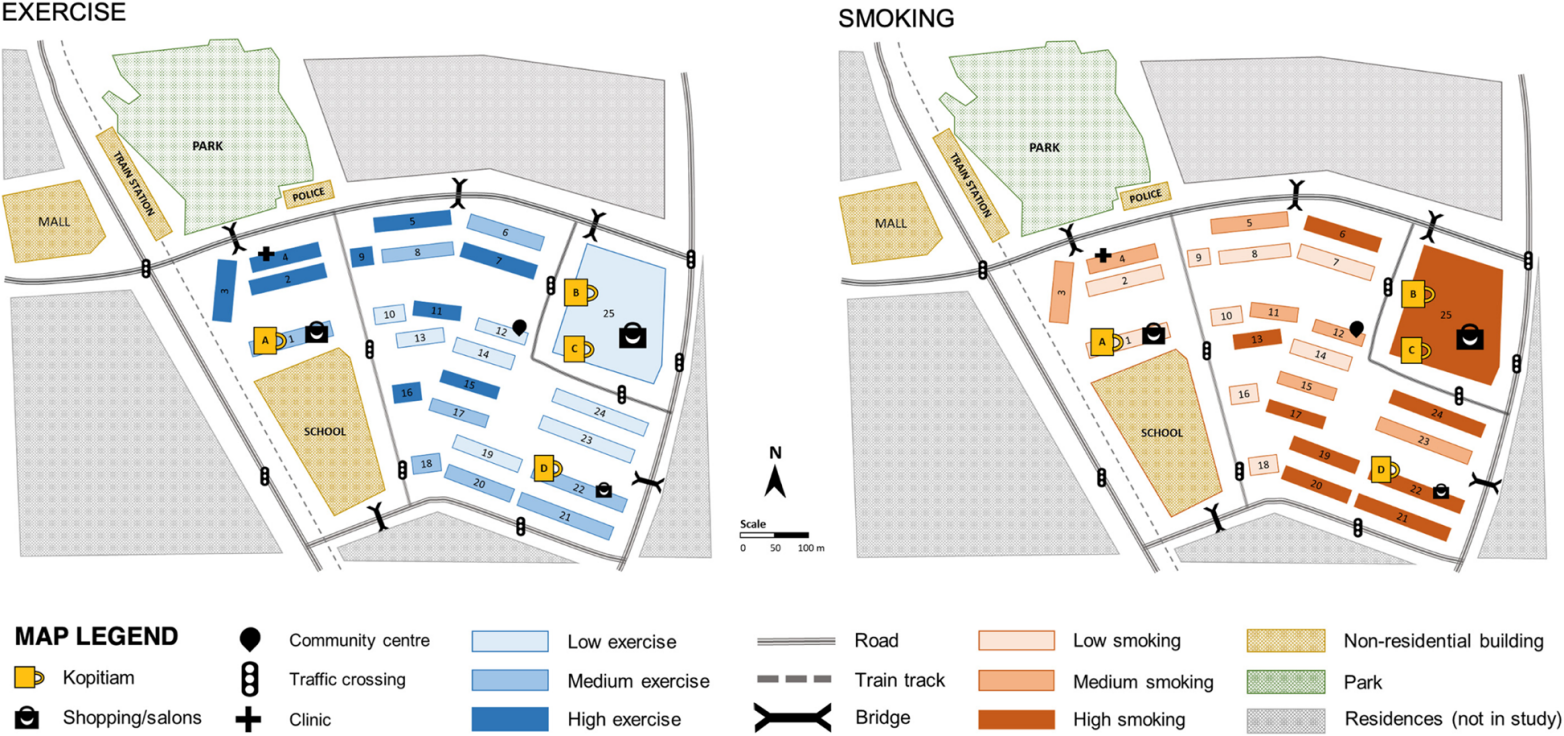
Neighborhood map colored by exercise and smoking. All blocks are ranked based on the prevalence of exercise and smoking among the participants. The rankings were then classified into approximate tertiles: low (< 1/3), medium (1/3 < 2/3), and high (> 2/3)

This study was part of a larger community-based participatory study of social connectedness and health among community-dwelling older adults in Singapore. The study was approved by the university research ethics committee. Located in the northeastern region of Singapore, the study town is the country’s seventh HDB public housing estate, making it one of the oldest towns [86]. It is officially classified as a mature town with a high concentration of older adults. There was a total of 32 residential blocks, including 24 high-rise and 8 low-rise buildings, all of which were approximately 40 years old. As of 2018, 42.5% of the study district residents are aged 55 and above [87]. As a self-sustained public housing estate, all the essential living facilities are available within the town, including an MRT (Mass Rapid Transit) train station nearby. With the recent urban renewal plan, an urban park was established right outside the town on the north-western edge of the town, which is connected to an MRT station and blocks by overhead bridges with lift access. A community active aging community center was established in 2019 to provide integrated community-based social and health care for older adults in the center of the town. Across the neighborhood, four *kopitiams* have been placed on the ground floor since the beginning of the town. Short traveling distances in the town with reasonable walkability of about 1 km allowed us to examine the effects of small-scale distance variation on older adults. Figure 1 shows the study neighborhood.

### Mixed methods design

We used a concurrent embedded mixed methods design where our ethnographic assessment was nested within a largely quantitative study, providing a supplemental role in regression analysis of the relationship between geographical proximity to parks and local coffeeshops and health behaviors [88]. We first conducted a door-to-door survey in all the blocks among older adults aged 60 or above in collaboration with a community organization running the activity center. A letter of the survey invitation was posted in the blocks a week before the data collection. During the verbal informed consent, we briefly assessed cognitive ability by whether they comprehended the study properly. The respondents received a voucher that could be exchanged for an umbrella to compensate for their time for the survey. A total of 497 older adults participated in the survey. The eight low-rise (about three stories) residential blocks have commercial stores on the first story and flats on the upper stories, and these blocks were combined into one block in analysis. Therefore, a total of 25 individual blocks were used in analysis. Across 25 blocks, a relatively equal number of participants were interviewed, with a range of 3 to 7% per block.

Concurrently, as a form of rapid ethnographic assessment, we first conducted social mapping and documented key features of the neighborhood in spatial domains: locations of social places (e.g., void decks on the ground floor of the blocks, playground, and park, coffeeshops, barbershops, and informal gathering places near to wet markets), community facilities (e.g., senior community and childcare centers), health services (e.g., primary care and traditional Chinese medicine clinics), and marketplaces [89, 90]. Based on the mapping, we conducted ethnographic observation to explore the social-cultural components of the neighborhood features, particularly the urban park and *kopitiams*. While observing how older adults got around the neighborhood and the kinds of interactions that took place in public spaces, we conducted “go-along” informal interviews with community workers and key informants (e.g., senior volunteers) to understand how older adults used the neighborhood places and what meanings were attached to the places [91]. Data were also collected in the form of field notes (e.g., informal conversation without taking direct quotes from participants) and photographs with permission. The findings of our formative research of various ethnographic assessments were used to develop interview guides in the subsequent phase of in-depth interviews with older adults. In the current study, ethnographic data were mainly used to contextualize the quantitative findings.

### Measures

#### Walking distance

We calculated the physical distance to the facilities of the park and *kopitiams* based on the residential blocks in which respondents resided. We mapped the places and referenced publicly available data on Google Earth to produce a neighborhood map. We observed the mobility paths to the places during our observation. On the Google Earth map, we also drew paths from the center of each block to the park and *kopitiams,* producing estimates of walking distances. This can better represent the respondents’ walking patterns than measuring straight-line distances. Older adults used traffic lights, zebra crossings, and sheltered walkways due to Singapore’s typically tropical weather. They also avoided car parks for safety. There was also a school at the western edge, which residents could not cut across. The calculated actual walking distances were checked against our observations.

#### Outcome variables of exercise and smoking

Physical activity was assessed by the frequency and intensity of exercise. For older adults, regular exercise was found to be more important than intensity in the reduction of mortality [92]. A 12-year prospective cohort study with older adults found that half an hour of light physical activity per 6 days a week was associated with about 40% risk reduction of mortality [93]. The importance of daily light exercise has been adopted by Health Promotion Board for older adults in Singapore [94]. In the study, physical activity was assessed using the explicit term of exercise: “how many days per week do you exercise?” This term implied an intention to increase fitness, distinguished from general activity accrued from other daily activities like grocery shopping. The responses deviated from the normal distribution, so we categorized them into the following three levels: none (0 days/week), some days (1–6 days/week), and every day (7 days/week). As an ordinal variable, the outcome variable of exercise consisted of three ordered categories that were ranked but not evenly spaced like interval data. Smoking was assessed as a binary variable: “do you smoke daily?” with a yes or no. Due to the small sample size of smoking older adults, we focused on daily habitual smoking instead of the variation in the frequency of smoking.

#### Covariates

In selecting the variables in our regression models, we conducted a literature review and identified control variables that were known to be associated with outcome variables. Physical activity was associated with individual characteristics [95], social support [96], social engagement at a larger social network like community attachment [97], and mental health [98]. The pathway from physical activity to mental health among older adults was explained by social support networks [99] and social relations [100]. In regard to smoking, compared to the disease consequences and benefits of smoking cessation [101], there is a dearth of data on social-behavioral factors associated with smoking among older adults. A recent study found that social isolation increased the risk of smoking [102].

In the study, potential covariates of exercise and smoking were demographic characteristics, physical and mental health status, social networks, and community attachment. Respondents were asked to indicate the number and types of chronic diseases they had. Mental health was assessed using the General Health Questionnaire (GHQ-12), a screening instrument for short-term psychiatric disorders widely used in population-based studies [103]. It consists of 12 items covering domains of anxiety and depression, social dysfunction, and loss of confidence. A unidimensional single-factor scale of the General Health Questionnaire was used to control for a confounding effect [104] and was highly reliable in the sample (Cronbach alpha = 0.89). The Lubben Social Network Scale (LSNS-6) [105] was used to measure the number of friends and family they interact with. The scale provided a good gauge of social support networks and was reliable in the sample (Cronbach alpha = 0.79). Community attachment was assessed using the Community Integration Measure [106], consisting of four domains: assimilation, support, occupation, and independent living. The scale was reliable in the sample (Cronbach alpha = 0.79).

### Statistical analysis

Univariate binary logistic regression models were used to examine the relationships between each of the measures (e.g., demographic characteristics and covariates) and each of the outcome variables (i.e., exercise represented by two binary variables: some days vs. none, and every day vs. none; and smoking: yes vs. no; some days, every day and yes were coded as 1, and none and no were coded as 0). In the analysis of the effects of physical distance, we mapped the rates of exercise and smoking of each block and the relationships with walking distances to each facility. The distance variable to each respondent’s data based on the blocks they resided in was calculated. Spearman rank correlations were used to examine bivariate associations between the study variables. Multiple regressions were conducted using the variables that were found to be significant at the univariate level; multicollinearity was tested using the Generalized Variance Inflation Factor. For exercise, hierarchical ordinal logistic regression models were performed, and the Brant test was used to assess the parallel regression (or proportional odds) assumption (see Supplementary Tables S1). When the assumption was found to be inadequate for any of the models, hierarchical multinomial logit regression models were performed instead to facilitate comparisons between the models. For smoking, hierarchical binary logistic regression models were performed. The hierarchical regression models consisted of demographic characteristics (step 1), covariates (step 2), and built environment composition – distance from the park and nearest *kopitiam* (step 3). In regression analysis, the distances were scaled to 100 m units for easier interpretation of results. For example, 943 m was scaled to 9.43 × 100 m. Adjusted OR (adj. OR) and 95% confidence intervals with model fit index were reported.

## Results

### Sample characteristics by exercise and smoking

The majority (85%) were aged from 60 to 80; 57% were females; 80% had at least primary school education; and 84% were unemployed or retired. More than half (63%) had at least one chronic disease. About one-third (*n* = 182, 37%) exercised every day, 41% (*n* = 202) did some days of the week, and 23% (*n* = 113) did not exercise. The minority (*n* = 50, 10%) smoked. Table 1 presents the participants' characteristics by exercise and smoking. A few demographic characteristics were associated with exercise. Compared with participants aged 60 to 70, the age group of 71 to 80 was associated with daily exercise only (OR = 2.33, *p* < 0.01). Compared with those who did not attend school, university education attainment was associated with exercise on some days (OR = 5.81, *p* < 0.01) and every day (OR = 4.48, *p* < 0.01), while secondary education attainment was associated with exercise on some days only (OR = 1.94, *p* < 0.05). Compared with those living in 3 rooms, living in 4 or 5 rooms was associated with daily exercise only (OR = 2.04, p < 0.01). With respect to psychosocial domains, more social networks (some days: OR = 1.11, *p* < 0.001; every day: OR = 1.09; *p* < 0.001) and community attachment (some days: OR = 1.14, *p* < 0.001; every day: OR = 1.12, *p* < 0.001) were associated with higher odds of exercise on some days and every day, while more psychological distress was associated with lower odds of daily exercise only (OR = 0.89, *p* < 0.001). Women were less likely to smoke than men (OR = 0.02; *p *< 0.001), and adults with 1–2 chronic diseases were more likely to smoke than other groups with morbidity (OR = 2.31; *p* < 0.05). Compared with older adults living 0–9 years in the neighborhood, no other groups living longer than nine years were associated with smoking or exercise. No significant association was found between exercise and smoking.

**Table 1:** Participants’ characteristics and univariate binary logistic regression for exercise and smoking

	Total	Exercise: *Some days vs. none*^*#*^	Exercise: *Everyday vs. none*^*#*^	Smoking: *Yes vs no*^*#*^
	N (%) or M (SD) ^	OR (95% CI)	OR (95% CI)	OR (95% CI)
Age				
60 – 70	229 (46.1%)	–	–	–
71 – 80	197 (39.6%)	1.61 (0.95–2.71)	2.33 (1.35–3.99)**	0.79 (0.42–1.48)
81 – 100	71 (14.3%)	0.52 (0.26–1.04)	1.19 (0.62–2.27)	0.44 (0.13–1.19)
Gender				
Male	214 (43.1%)	–	–	–
Female	283 (56.9%)	0.86 (0.54–1.38)	0.74 (0.46–1.20)	0.02 (0.00–0.08)***
Education				
Did not attend	99 (19.9%)	–	–	–
Primary	171 (34.4%)	1.61 (0.86–3.02)	1.21 (0.65–2.25)	2.17 (0.89–6.09)
Secondary	166 (33.4%)	1.94 (1.02–3.67)*	1.50 (0.80–2.83)	1.89 (0.76–5.35)
University	61 (12.3%)	5.81 (2.00–16.91)**	4.48 (1.53–13.07)**	1.38 (0.38–4.80)
Working status				
Employed	80 (16.1%)	–	–	–
Unemployed	417 (83.9%)	1.43 (0.80–2.58)	1.78 (0.95–3.31)	0.65 (0.33–1.38)
Residence years				
0–9 years	40 (8.0%)	-	-	-
10–19 years	65 (13.1%)	2.20 (0.74–6.53)	0.86 (0.29–2.57)	0.85 (0.25–3.04)
20–29 years	93 (18.7%)	1.45 (0.54–3.93)	0.67 (0.25–1.78)	1.04 (0.35–3.50)
30–39 years	103 (20.7%)	1.15 (0.43–3.05)	0.65 (0.25–1.69)	0.59 (0.18–2.06)
40–49 years	196 (39.4%)	1.01 (0.40–2.55)	0.90 (0.38–2.17)	0.71 (0.26–2.26)
Flat type				
3 room	303 (61.0%)	–	–	–
4 or 5 room	194 (39.0%)	1.37 (0.84–2.25)	2.04 (1.24–3.34)**	0.58 (0.29–1.08)
Multimorbidity				
None	184 (37.0%)	–	–	–
1 or 2	147 (29.6%)	1.12 (0.63–1.97)	1.12 (0.63–1.99)	2.31 (1.14–4.89)*
3 or more	166 (33.4%)	1.27 (0.73–2.20)	1.15 (0.66–2.02)	1.30 (0.60–2.87)
Psychological distress^	22.1 (4.40)	0.97 (0.92–1.01)	0.89 (0.84–0.94)***	1.03 (0.96–1.09)
Social networks^	13.5 (6.24)	1.11 (1.07–1.16)***	1.09 (1.04–1.13)***	0.99 (0.94–1.03)
Community attachment^	26.3 (3.78)	1.14 (1.06–1.21)***	1.12 (1.05–1.20)***	0.96 (0.89–1.04)
Exercise				
Never	113 (22.7%)			–
Some days	202 (40.6%)			0.84 (0.50–1.47)
Everyday	182 (36.7%)			1.04 (0.64–1.72)
Smoking	50 (10.1%)	0.85 (0.40–1.77)	0.79 (0.37–1.70)	

Psychological distress (General Health Questionnaire-12): range 12–48. Social networks (Lubben Social Network Scale-6): range 0–30. Community attachment (Community Integration Measure): 9–36. #: Some days, every day and yes were coded as 1, and none and no were coded as 0

^*^ *p* < 0.05

^**^ *p* < 0.01

^***^ *p* < 0.001

### Spatial distribution of exercise and smoking

Figure 1 shows the distribution of the rates of exercise and smoking across each block and the locations of the urban park and *kopitiams* in the neighborhood. The three levels of rates were calculated based on ranked order. Higher rates of exercise were reported in the residential blocks on the west side of the town, where the park was located across the street. In contrast, higher rates of smoking were clustered in the blocks on the east side of the town, where three *kopitiams* were located. To check whether there are parks and kopitiams in other directions, a map showing the surrounding areas of the study neighborhood is provided in a supplementary file (see Supplementary Figure S1). There are two parks on the northwest and southwest sides of the neighborhood. However, the study neighborhood is bounded by large roads without shelters. Within the neighborhood, most walkways are provided with shelters, which protect pedestrians from heat and rain (e.g., frequent showers). Those two parks are relatively far and less accessible for older adults in the study neighborhood. While there are kopitiams on the west sides of the neighborhood, it is highly unlikely for older adults to visit the places and spend their daily time except on special occasions. As described earlier in the introduction, public housing estates are built as self-sustained towns providing essential facilities, primary healthcare, wet markets, and kopitiams for the residents of the belonging estate.

**Fig. 2:**
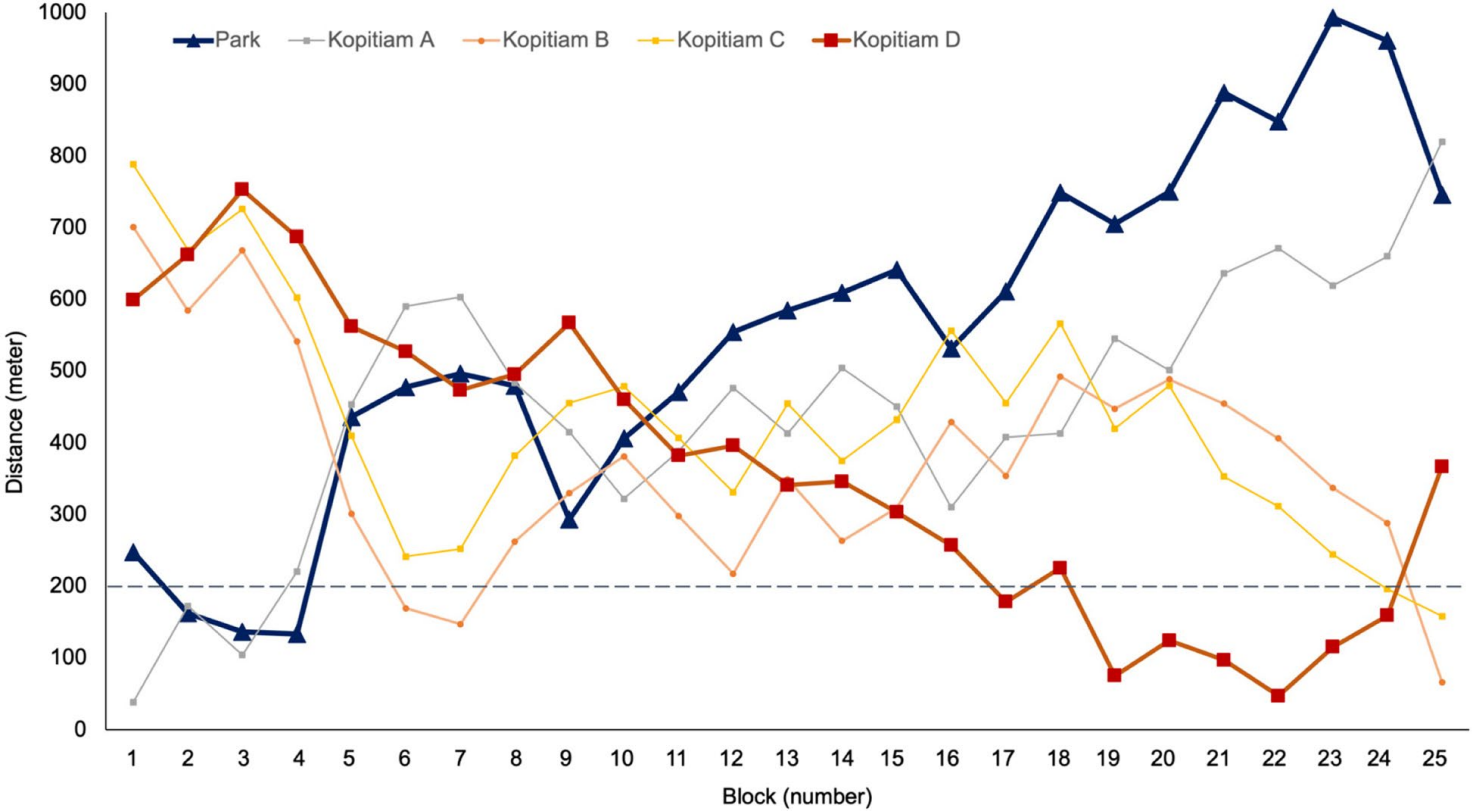
Walking distance between residential blocks and the neighbourhood facilities: an urban park and four kopitiams (traditional local coffeeshop). On the x-axis of blocks, blue denotes high rate of exercise and red denotes high rate of smoking. On the x-axis of blocks, the blocks reporting high rates of exercise are 2,3,4,5,7,9,11,15, and 16; and high rates of smoking are 6,13,17,19,20,21,22,24, and 25

Figure 2 shows the walking distance from the residential blocks to the facilities. The numbering of the blocks runs from west to east, as presented in Fig. 1. The blocks located close to the park, with a walking distance below 200 m (e.g., blocks 2–4), reported high rates of frequent exercise. The clustering pattern of smoking was more noticeable than that of exercise. Overall, while four *kopitiams* (traditional local coffeeshops) were scattered in the town within a 400 m walking distance of each block, the blocks located within walking distance of 200 m from *kopitiam* reported a high rate of smoking, in particular from *kopitiam* B, which was located in the east end of the town (e.g., blocks 17–24), the furthest from the park. Those blocks near *kopitiam* B are 600 m walking distance from the park.

### Correlations

Table 2 shows Spearman correlations between key variables. Psychological distress was negatively correlated with social networks (*r* = -0.12) and community attachment (*r* = -0.21). Social networks and community attachment were positively correlated (*r* = 0.43). Distance from the park was positively correlated with multimorbidity (*r* = 0.15), psychological distress (*r* = 0.15), and smoking (*r* = 0.14), which was negatively correlated with the distance from *kopitiam* (*r* = -0.10). Exercise was moderately correlated with all the covariates of psychological distress, social networks, and community attachment (range *r* = 0.12 to 0.14). No correlation was found between exercise and smoking. Multimorbidity was not correlated with psychological distress, social networks, community integration, kopitiam distance, exercise, or smoking. Psychological distress was not correlated with kopitiam distance or smoking. Social network was not correlated with kopitiam and park distance or smoking. Community integration was not correlated with kopitiam and park distance or smoking. The two outcome variables of exercise and smoking were not correlated with each other.

**Table 2:** Spearman correlations between key variables

	1	2	3	4	5	6	7	8
1. Multimorbidity	-							
2. Psychological distress	.08	-						
3. Social networks	.02	-.12**	-					
4. Community integration	-.01	-.21***	.43***	-				
5. Kopitiam distance	-.03	.00	-.01	.04	-			
6. Park distance	.15***	.21***	.01	-.03	-.22***	-		
7. Exercise	.02	-.14**	.13**	.12**	.11*	-.17***	-	
8. Smoking	.03	.03	-.03	-.05	-.10*	.14**	-.03	-

Kopitiam (traditional local coffeeshop) distance was calculated from the nearest Kopitiam

^*^ *p* < 0.05

^**^ *p* < 0.01

^***^ *p* < 0.001

### Regressions for exercise and smoking

The hierarchical regression models for exercise and smoking are presented in Tables 3 and 4, respectively. The following variables remained as independent significant factors at the final step of the regression model. Older adults aged 71 to 80 had higher odds of daily exercise than those aged 60 to 70 (adj. OR = 2.44, 95% CI: 1.37–4.34). Those with university education had higher odds of exercise on some days (adj. OR = 4.33, 95% CI: 1.39–13.53) and every day (adj. OR = 3.33, 95% CI: 1.05–10.54) when compared to those who did not attend school. With respect to psychosocial domains, more psychological distress was significantly associated with lower odds of daily exercise only (adj. OR = 0.92, 95% CI: 0.86–0.98), while more social networks were significantly associated with higher odds of exercise on some days (adj. OR = 1.09, 95% CI: 1.04–1.14) and every day (adj. OR = 1.06, 95% CI: 1.01–1.11). For built environment factors, there was a borderline significance (*p* = 0.052) between longer walking distance from the urban park and lower odds of daily exercise (adj. OR = 0.90, 95% CI: 0.82–1.00).

**Table 3:** Hierarchical multinomial logit regression for exercise

	Step 1	Step 2	Step 3
	Adj. OR (95% CI)	Adj. OR (95% CI)	Adj. OR (95% CI)
***Exercise: Some days vs. none***			
**1. Demographic**			
Gender			
Male	–	–	–
Female	0.93 (0.57– 1.52)	0.82 (0.49 -1.38)	0.84 (0.50–1.41)
Age			
60 – 70	–	–	–
71 – 80	1.74 (1.02–2.97)*	1.78 (1.03–3.08)*	1.71 (0.99–2.99)
81 – 100	0.66 (0.32–1.38)	0.64 (0.30–1.37)	0.59 (0.27–1.27)
Education			
Did not attend school	–	–	–
Primary school	1.39 (0.72–2.69)	1.33 (0.66–2.65)	1.36 (0.68–2.74)
Secondary school	1.67 (0.85–3.29)	1.62 (0.80–3.30)	1.59 (0.78–3.26)
University	4.82 (1.59–14.66)**	4.31 (1.38–13.41)*	4.33 (1.39–13.53)*
Flat type			
3-room	–	–	–
4/5-room	1.25 (0.75–2.10)	1.23 (0.72–2.10)	1.01 (0.56–1.83)
**2. Health status**			
Multimorbidity			
None	–	–	–
1 or 2		1.16 (0.63–2.12)	1.18 (0.65–2.17)
3 or more		1.32 (0.73–2.38)	1.41 (0.77–2.58)
Psychological distress		1.00 (0.95–1.06)	1.01 (0.95–1.06)
Social networks		1.09 (1.04–1.14)***	1.09 (1.04–1.14)***
Community attachment		1.07 (0.99–1.15)	1.07 (0.99–1.15)
**3. Built environment**			
Park distance			0.95 (0.86–1.06)
Kopitiam distance			1.21 (0.89–1.62)
***Exercise: Every day vs. none***			
**1. Demographic**			
Gender			
Male	–	–	–
Female	0.78 (0.48–1.30)	0.66 (0.39–1.12)	0.67 (0.40–1.14)
Age			
60 – 70	–	–	–
71 – 80	2.57 (1.48–4.47)***	2.60 (1.47–4.59)***	2.44 (1.37–4.34)**
81 – 100	1.39 (0.69–2.81)	1.36 (0.66–2.84)	1.23 (0.59–2.58)
Education			
Did not attend school	–	–	–
Primary school	1.14 (0.59–2.23)	1.04 (0.52–2.10)	1.11 (0.54–2.25)
Secondary school	1.43 (0.72–2.83)	1.37 (0.68–2.82)	1.34 (0.65–2.77)
University	3.82 (1.24–11.82)*	3.29 (1.04–10.35)*	3.33 (1.05–10.54)*
Flat type			
3-room	–	–	–
4/5-room	1.91 (1.13–3.20)*	1.75 (1.03–3.00)*	1.30 (0.72–2.36)
**2. Health status**			
Multimorbidity			
None		–	–
1 or 2		1.25 (0.68–2.32)	1.28 (0.69–2.37)
3 or more		1.25 (0.68–2.89)	1.39 (0.75–2.59)
Psychological distress		0.91 (0.85–0.97)**	0.92 (0.86–0.98)*
Social networks		1.06 (1.01–1.11)*	1.06 (1.01–1.11)*
Community attachment		1.04 (0.96–1.13)	1.04 (0.97–1.13)
**3. Built environment**			
Park distance			0.90 (0.82–1.00)
Kopitiam distance			1.21 (0.89 – 1.64)
McFadden’s Pseudo R^2^	0.036	0.078	0.084
∆ R^2^	–	0.042	0.006

Kopitiam (traditional local coffeeshop) distance was calculated from the nearest Kopitiam

^*^ *p* < 0.05

^**^ *p* < 0.01

^***^ *p* < 0.001

**Table 4:** Hierarchical binary logistic regression for smoking

	Step 1	Step 2	Step 3
	Adj. OR (95% CI)	Adj. OR (95% CI)	Adj. OR (95% CI)
**1. Demographic**			
Gender			
Male	–	–	–
Female	0.02 (0.01–0.07)***	0.02 (0.01–0.07)***	0.02 (0.01–0.05)***
Age			
60 – 70	–	–	–
71 – 80	0.64 (0.32–1.27)	0.69 (0.34–1.37)	0.76 (0.36–1.57)
81 – 100	0.31 (0.08–0.91)*	0.24 (0.06–0.72)*	0.21 (0.05–0.71)*
Education			
Did not attend school	–	–	–
Primary school	0.98 (0.34–3.07)	0.81 (0.28–2.59)	0.59 (0.18–2.01)
Secondary school	0.93 (0.32–2.98)	0.78 (0.26–2.54)	0.81 (0.25–2.82)
University	0.52 (0.12–2.09)	0.41 (0.09–1.72)	0.34 (0.07–1.58)
Flat type			
3-room	–	–	–
4/5-room	0.73 (0.35–1.50)	0.70 (0.32–1.49)	1.52 (0.62–3.80)
**2. Health status**			
Multimorbidity			
None		–	–
1 or 2		2.94 (1.31–6.86)*	3.57 (1.51–8.89)**
3 or more		1.27 (0.55–3.01)	1.00 (0.40–2.49)
Psychological distress		0.96 (0.89–1.03)	0.93 (0.86–1.00)
Social networks		1.01 (0.95–1.07)	1.00 (0.94–1.06)
Community attachment		0.96 (0.86–1.07)	0.95 (0.85–1.06)
**3. Built environment**			
Park distance			1.28 (1.11–1.50)**
Kopitiam distance			0.59 (0.37–0.91)*
McFadden’s Pseudo R^2^	0.249	0.274	0.328
∆ R^2^	–	0.025	0.054

Kopitiam (traditional local coffeeshop) distance was calculated from the nearest Kopitiam

^*^ *p* < 0.05

^**^ *p* < 0.01

^***^ *p* < 0.001

In the final step of regression for smoking, adults in their age of over 80 years old and females were less likely to smoke than their counterparts (adj. OR = 0.21, 95% CI: 0.05–0.71 and adj. OR = 0.02, 95% CI: 0.01–0.05, respectively). Compared to adults with no chronic disease, those with 1–2 chronic diseases had significantly higher odds of smoking (adj. OR = 3.57, 95% CI: 1.51–8.89). The walking distances from both facilities of the park and *kopitiams* were significantly associated with smoking. The odds of smoking decreased with an increase in distance from *kopitiams* (OR = 0.59, 95% CI: 0.37–0.91). Conversely, the odds of smoking increased with an increase in distance from the park (adj. OR = 1.28, 95% CI: 1.11–1.50).

As shown in Fig. 1, the relationship between proximity to the urban park and *kopitiams* and exercise frequency was confounded by other factors, such as social networks. For example, there are blocks 7,11, and 15 located near *kopitiams* but have high levels of exercise. A post-hoc analysis, using ANOVA, was conducted to compare social networks between the three groups of blocks: (1) blocks near *kopitiams* with high-level of exercise (blocks 7,11, and 15; *n* = 47), (2) blocks near *kopitiams* with high-level of smoking (blocks 6, 12, 13, 14, 17, and 19 to 25; *n* = 125), and (3) other blocks neither near *kopitiams* or high level of smoking (all other blocks; *n* = 325). There was a significant difference across the three groups (F = 4.442, *p* = 0.012). The blocks in groups 1 and 3 reported higher mean scores on social networks (Group 1: Mean = 14.8, SD = 5.5; Group 3: Mean = 13.8, SD = 6.5) than those in group 2 (M = 12.2, SD = 5.7). Older adults living in blocks near kopitiams with high-level of smoking reported lower social networks.

## Discussion

The study indicated the effects of neighborhood features on exercise and smoking among older adults in a public housing town in Singapore. To the best of our knowledge, this is the first study that investigated the spatial relationship between *kopitiams* and health behaviors. The findings indicated that overall, older adults living near the urban park exercised more and smoked less than those living far from the park. Social networks were found to play a key role in exercise among older adults. In contrast, older adults living near a *kopitiam* smoked more. The physical distances to the facilities were short, about 200 m in walking distance. The finding that even a couple of blocks yielded observable effects on behaviors suggests that older adults’ healthy lifestyles are closely affected by their physical boundaries of the neighborhood places. The clustered effects of exercise and smoking by the neighborhood features among older adults, in particular among males, are notable; further research is warranted in terms of how these urban and traditional places will influence the formation of social identity and norms in later life.

The finding of the spatial relationship between the park and exercise is consistent with previous studies reporting that living near parks increased exercise frequency after controlling for demographic and health variables [47, 107]. The findings that proximity to the park decreased the odds of smoking can be explained by attitudinal and behavioral aspects. Smoking is prohibited in urban parks in the country. The environmental constraint for smoking facilitates health communication of the social disapproval of smoking [108]. While an indirect effect on smoking cessation was expected, there was a correlational relationship between exercise and smoking in our sample of older adults who might be introduced to exercise while having a long-term habit of smoking. The finding was consistent with a systemic review of the relationship between physical activity and smoking that about 60% of reviewed studies reported a direct negative association between the two behaviors, while the rest showed potential moderating factors of demographic characteristics, including age, gender, ethnicity, marital status, and employment status [68], and other empirical studies reporting the linkages between two behaviors among older adults as described in the introduction [74]. The complexity of incongruent practices of exercise and smoking among older adults, perhaps long-term habitual smokers, requires further research for intervention.

Gendered place attachment and gender roles in social and community participants among older adults are important in healthy aging. We conducted a post-hoc analysis to explore the gender role in the outcome variables by splitting the sample by gender. With respect to smoking, only two women reported smoking; therefore, post-hoc analysis was not possible. In the sample of males, physical distance to kopitiam remained one of the independent factors for smoking. As discussed below, kopitiams were occupied mainly by males; many of whom were smokers. With respect to exercise, gender effects are apparent in terms of proximity to the urban parks and kopitiams. For males, the close proximity to kopitiams was significantly associated with lower odds of exercise on some days and every day (see Supplementary Table S2). Older men who lived in the blocks located further from kopitiams engaged in exercise more frequently than those living in the blocks nearby. For females, while the longer distance from the park appeared to be associated with lower odds of exercise on every day at a marginal significance level (adj. OR = 0.88, 95% CI: 0.77–1.00; *p* = 0.057; see Supplementary Table S3), social networks were found to be more important in promoting exercise. Our ethnographic observation and interviews with community organizations supported this finding. Older women in the study community participated in a wider range of leisure activities on a general level. Aside from visiting the park for exercise, they engaged in group physical activities from other sources, such as exercise at the void deck as a group and events held by our partner community organization. The study findings suggest that place attachment and utilization of community sources would result in gender discrepancy in healthy aging.

The finding of clustering smoking older adults by *kopitiams* highlights the social-cultural and environmental roles of the communal place in healthy aging in place. As described, *kopitiams* represents an everyday site of meals, gatherings, and a source of gossip and stories of the community since early colonial Singapore. Safeguarded as part of the nation’s heritage as “community dining rooms” [109], the traditional coffeeshops have been focal points of urban life. The literature of environmental gerontology emphasized the importance of the role of communal places, like *kopitiams*, among older adults, as a place of social relation formation and maintenance [45], “public familiarity with familiar strangers” [110] and “third places” [17]. During our ethnographic assessment, older adults, particularly males, expressed *kopitiams* as the most meaningful places for their everyday life. Social interactions at *kopitiams* were very casual and fluid, where they built intimate relationships with the owners and were open to sharing tables with strangers and engaging with them. The personal boundary was rarely observed where older adults engaged in conversations that seemed not orchestrated, controlled, or operated by any order. There was constant and spontaneous sharing of what was going on in the community and how others were getting along. Regulars who sat without time restrictions at the coffeeshops became “eyes on the street” in mutual surveillance, yet their activities did not have to be “meaningful,” such as having quiet moments to oneself and people watching [111].

*Kopitiams* are marked as a core of informal social ties. Older adults’ daily visits provide them with agency to transform the place into a “home in my neighborhood” where they feel attached, included, supported, and secured. Their narratives of interpersonal relations inside and around the casual and multigenerational settings of the *kopitiams* – older adults’ sharing tables spontaneously with strangers and friends and greeting other passing-by neighbors – construct individual and collective identity of older adults in the community. During our fieldwork, there were existential meanings attached to the coffeeshop, where older adults came and shared their stressful life events with friends over affordable food and drinks. Conversations revolve around conflicts with neighbors, health issues, home maintenance, adult children moving out, retirement, and bereavement of family and friends. While experiencing neighborhood changes and life transitions in older age, older adults attempted to preserve memories and the symbolic identity of the neighborhood by collective remembering through *kopitiams*. Older adults’ stories heard from the coffeeshops allowed us to better understand the context and history of the neighborhood.

Nonetheless, there is a conflicting role of *kopitiams* in physical and social well-being for smoking older adults (mostly males). Smoking is strictly prohibited in public places by law. While neighborhood places of bonding facilitate social connectedness, a *kopitiam*, known to be a place of gathering for smoking and social leisure for males, still allows smoking as exceptional from the policy of smoking ban in the public place. Places for social life are limited for older adults, particularly smoking adults. Although there are community centers for older adults, smokers are not allowed. Smoking older adults did not want to “feel bothered” by anti-smoking messages. For example, we observed mostly female older adults attending the community center in block 12 across *kopitiams*. For smoking older adults, smoking is habitual as part of their daily routine. The coffeeshops provide space for mingling while preserving the cultural meanings of ‘good old days (with smoking).’ There was a permissible norm for smoking and no intention of quitting smoking from the smoking older adults we have had conversations with.

The findings of the variability in the smoking rates of residential blocks across *kopitiams* suggested that the dynamics of social interactions within a coffeeshop and between a coffeeshop and other places in surrounding areas could play a role in clustered smoking. We observed that the smoking corner was predominantly occupied by groups of older men at *kopitiam* D in the middle of the town, purchasing drinks and cigarettes from a beverage stall. In return for their patronage, they continued chatting at the tables for hours while smoking. Neighbor older adults who were passing by occasionally joined the table to chat. Unlike *kopitiam* D, *kopitiam* A was located near the commercial places of the mall, MRT station, and school. It had different compositions of human traffic and smoking behaviors. Although the physical features were similar to other coffeeshops, the smoking area was occupied mainly by younger men and office workers who stopped by for a quick lunch break. We rarely observed a group of smoking older adults sitting there for a long time. The different demographic patterns of socialization at *kopitiam* might explain low rates of smoking among older adults from the north-western cluster of blocks 1–4, which were instead reported high rates of exercise.

Older adults are limited in accessing public places for social connections. More places with walkability are necessary for older adults to engage in the community actively. This will achieve healthy aging in place. With more public housing towns being due for urban renewal with the introduction of urban features, socioecological intervention is required to promote better adaptation among older adults who have lived in the same neighborhood for decades. It requires beyond the mere provision of facilities. Older adults’ subjective meanings and emotional attachment to communal places are more important. Their agency in selecting diverse places for their social life is crucial in policy decision-making.

### Limitations

A small sample size from a moderately low (60%) response rate could pose a bias that decreased the reliability and external validity of the results. We visited older adults’ homes twice to reduce the chance where non-responses occurred when older adults were not present to participate in our survey. The self-reported measures of physical activity might increase a potential recall bias of the accuracy of the assessment. The rate of smoking older adults in the sample was 10.1% (n = 50, including two females). This was consistent with the smoking prevalence of 10.1% from the National Population Health Survey (NPHS) in 2020 [112]. While we focused on the differences between smoking and non-smoking older adults, such a binary measure was less ideal for assessing the factors associated with smoking frequency. Large samples of smoking older adults would be necessary to better understand smoking behaviors among older adult populations. The study community is a mature estate. All the *kopitiams* in the neighborhood remained traditional. Modern *kopitiams* in new public housing estates might not serve the same roles for older adults. *Kopitiams* were predominately occupied by older male adults. Female older adults were more seen at indoor activities within community centers. A local study reported that closer proximity to traditional wet markets was associated with better social health [113]. Gender roles in place attachment among older adults need further exploration. The study focused on two facilities. Other facilities in the neighborhood could affect health behaviors among older adults, such as open spaces for social gatherings and exercise like *qigong* in the void decks of housing on the ground floor. The study was conducted in October 2019 and halted in February 2020 due to the COVID-19 pandemic. Due to mobility restrictive measures, we could not continue to observe patterns of gathering in other places. Although we observed the behavioral patterns of smoking at *kopitiams* in the ethnographic phase, the study did not measure the time individual participants spent in the places. Thus, the relationship between distance and duration in the places is unknown. Geoinformation-technology based tools (e.g., GPS trackers) can be used to produce objective data on mobility among older adults. Social network data on exercise and smoking in the group will also identify the structure and positions of networks of exercising and smoking older adults.

## Conclusions

The neighborhood effects on health behaviors are neither unidirectional nor dichotomous. It is important to understand how the urban environment and older adults interact with each other to maintain and reconstruct the meaning of the places through reintegrating identity and attached meanings, where older adults gather and spend time in various age-friendly neighborhood places offered for them. The compositional and contextual attributes of neighborhood places are crucial in urban planning for healthy aging in place. The architectural and spatial layouts of urban features and traditional places for older adults will impact their health. Urban development and renewal have improved overall neighborhood safety and efficiency of access to essential services. However, the spaces for older adults’ social needs, like *kopitiams*, barbershops, traditional board and chess game houses, and old wet markets, are often overlooked, replaced, or relocated, although their daily lives and identity are more bounded by the neighborhood. Along with urban parks that promote physical activity, these social–historical spaces need to be provided within walking distance to promote psychosocial well-being, while appropriate interventions are necessary to reduce risk practices like smoking and habitual drinking among older adults. Creating meaningful places that meet the needs of older adults will achieve healthy aging in place.

## Supplementary Information

**Additional file 1:** **Table S1.** Brant test results on proportional odds models. **Table S1.1.** Brant test (Overall sample). **Table S1.2.** Brant test (Males: N=214). **Table S1.3.** Brant test (Females; N=283). **Table S2.** Hierarchical multinomial regression for exercise among males (N=214). **Table S3.** Hierarchical multinomial regression for exercise among females (N=283). **Figure S1.** Map of Surrounding Areas of the Study Neighborhood.

## Data Availability

The datasets generated during and analyzed during the current study are not publicly available due to the risk of identifying the participants but are available from the corresponding author upon reasonable request.

## Abbreviations

HDB: Housing and Development Board
UNESCO: United Nations Educational, Scientific and Cultural Organization
MRT: Mass Railroad Transit
COVID-19: Coronavirus Disease 2019
