# Supplementary material for: Effects of neighborhood features on healthy aging in place: the composition and context of urban parks and traditional local coffeeshops in Singapore
Source: BMC Geriatr. 2022 Dec 15;22:969. doi: 10.1186/s12877-022-03679-z (PMC9753030; doi:10.1186/s12877-022-03679-z)
Supplement: Supplementary file 1 — Additional file 1: Table S1. Brant test results on proportional odds models. Table S1.1. Brant test (Overall sample). Table S1.2. Brant test (Males: N=214). Table S1.3. Brant test (Females; N=283). Table S2. Hierarchical multinomial regression for exercise among males (N=214). Table S3. Hierarchical multinomial regression for exercise among females (N=283). Figure S1. Map of Surrounding Areas of the Study Neighborhood. [file 12877_2022_3679_MOESM1_ESM.docx]

**Supplementary Tables**

**Table S1. Brant test results on proportional odds models**

**Table S1.1. Brant test (Overall sample)**

|  | Step 1 | Step 2 | Step 3 |
| --- | --- | --- | --- |
|  | χ2 (df) | χ2 (df) | χ2 (df) |
| **1. Demographic** |  |  |  |
| Gender |  |  |  |
| Male | – | – | – |
| Female | 0.02 (1) | 0.01 (1) | 0.00 (1) |
| Age |  |  |  |
| 60 – 70 | - | - | - |
| 71 – 80 | 0.41 (1) | 0.43 (1) | 0.42 (1) |
| 81 – 100 | 3.50 (1) | 3.56 (1) | 4.07 (1)* |
| Education |  |  |  |
| Did not attend school | - | - | - |
| Primary school | 0.87 (1) | 0.64 (1) | 0.62 (1) |
| Secondary school | 1.54 (1) | 1.20 (1) | 1.10 (1) |
| University | 5.30 (1)* | 4.56 (1)* | 4.53 (1)* |
| Flat type |  |  |  |
| 3-room | - | - | - |
| 4/5-room | 0.09 (1) | 0.05 (1) | 0.19 (1) |
| **2. Health status** |  |  |  |
| Multimorbidity |  |  |  |
| None |  | - | - |
| 1 or 2 |  | 0.06 (1) | 0.11 (1) |
| 3 or more |  | 0.57 (1) | 0.68 (1) |
| Psychological distress |  | 3.93 (1)* | 3.89 (1)* |
| Social networks |  | 9.94 (1)** | 9.85 (1)** |
| Community attachment |  | 1.92 (1) | 1.87 (1) |
| **3. Built environment** |  |  |  |
| Park distance |  |  | 0.00 (1) |
| Kopitiam distance |  |  | 0.54 (1) |
| Omnibus | 15.14 (7)* | 40.11 (12)*** | 41.56 (14)*** |

Note: Kopitiam (traditional local coffeeshop) distance was calculated from the nearest Kopitiam. * p < 0.05; ** p < 0.01; *** p < 0.001.

**Table S1.2. Brant test (Males: N=214)**

|  | Step 1 | Step 2 | Step 3 |
| --- | --- | --- | --- |
|  | χ2 (df) | χ2 (df) | χ2 (df) |
| **1. Demographic** |  |  |  |
| Age |  |  |  |
| 60 – 70 | - | - | - |
| 71 – 80 | 0.16 (1) | 0.05 (1) | 0.04 (1) |
| 81 – 100 | 4.06 (1)* | 3.88 (1)* | 3.75 (1) |
| Education |  |  |  |
| Did not attend school | - | - | - |
| Primary school | 1.04 (1) | 0.67 (1) | 1.12 (1) |
| Secondary school | 2.64 (1) | 2.15 (1) | 2.37 (1) |
| University | 3.05 (1) | 2.01 (1) | 2.24 (1) |
| Flat type |  |  |  |
| 3-room | - | - | - |
| 4/5-room | 0.00 | 0.01 (1) | 0.01 (1) |
| **2. Health status** |  |  |  |
| Multimorbidity |  |  |  |
| None |  | - | - |
| 1 or 2 |  | 0.14 (1) | 0.16 (1) |
| 3 or more |  | 0.86 (1) | 1.05 (1) |
| Psychological distress |  | 0.17 (1) | 0.21 (1) |
| Social networks |  | 2.38 (1) | 2.27 (1) |
| Community attachment |  | 0.47 (1) | 0.54 (1) |
| **3. Built environment** |  |  |  |
| Park distance |  |  | 1.41 (1) |
| Kopitiam distance |  |  | 0.95 (1) |
| Omnibus | 12.49 (6) | 22.07 (11)* | 23.60 (13)* |

Note: Kopitiam (traditional local coffeeshop) distance was calculated from the nearest Kopitiam. * p < 0.05; ** p < 0.01; *** p < 0.001.

**Table S1.3. Brant test (Females; N=283)**

|  | Step 1 | Step 2 | Step 3 |
| --- | --- | --- | --- |
|  | χ2 (df) | χ2 (df) | χ2 (df) |
| **1. Demographic** |  |  |  |
| Age |  |  |  |
| 60 – 70 | - | - | - |
| 71 – 80 | 1.04 (1) | 0.75 (1) | 0.71 (1) |
| 81 – 100 | 0.73 (1) | 1.32 (1) | 1.65 (1) |
| Education |  |  |  |
| Did not attend school | - | - | - |
| Primary school | 0.59 (1) | 0.62 (1) | 0.53 (1) |
| Secondary school | 0.26 (1) | 0.14 (1) | 0.10 (1) |
| University | 3.02 (1) | 2.61 (1) | 2.55 (1) |
| Flat type |  |  |  |
| 3-room | - | - | - |
| 4/5-room | 0.28 (1) | 0.01 (1) | 0.23 (1) |
| **2. Health status** |  |  |  |
| Multimorbidity |  |  |  |
| None |  | - | - |
| 1 or 2 |  | 0.28 (1) | 0.33 (1) |
| 3 or more |  | 2.73 (1) | 2.98 (1) |
| Psychological distress |  | 10.78 (1)** | 10.76 (1)** |
| Social networks |  | 6.76 (1)** | 6.79 (1)** |
| Community attachment |  |  |  |
| **3. Built environment** |  |  | 0.00 (1) |
| Park distance |  |  | 0.64 (1) |
| Kopitiam distance |  |  |  |
| Omnibus | 7.24 (6) | 43.03 (11)*** | 43.94 (13)*** |

Note: Kopitiam (traditional local coffeeshop) distance was calculated from the nearest Kopitiam. * p < 0.05; ** p < 0.01; *** p < 0.001.

**Table S2. Hierarchical multinomial regression for exercise among males (N=214)**

|  | Step 1 | Step 2 | Step 3 |
| --- | --- | --- | --- |
|  | Adj. OR (95% CI) | Adj. OR (95% CI) | Adj. OR (95% CI) |
| ***Exercise: Some days vs. none*** |  |  |  |
| **1. Demographic** |  |  |  |
| Age |  |  |  |
| 60 – 70 | – | – | – |
| 71 – 80 | 1.40 (0.59 – 3.31) | 1.50 (0.61 – 3.69) | 1.50 (1.18 – 7.93) |
| 81 – 100 | 0.50 (0.17 – 1.52) | 0.46 (0.14 – 1.54) | 0.46 (0.13 – 1.57) |
| Education |  |  |  |
| Did not attend school | – | – | – |
| Primary school | 2.07 (0.61 – 7.04) | 1.85 (0.49 – 7.04) | 2.30 (0.58 – 9.08) |
| Secondary school | 2.54 (0.73 – 8.90) | 2.43 (0.61 – 9.66) | 2.49 (0.62 – 10.05) |
| University | 7.61 (1.17 – 49.39)* | 5.38 (0.75 – 38.59) | 5.77 (0.80 – 41.85) |
| Flat type |  |  |  |
| 3-room | – | – | – |
| 4/5-room | 1.29 (0.54 – 3.04) | 1.15 (0.46 – 2.89) | 0.95 (0.35 – 2.62) |
| **2. Health status** |  |  |  |
| Multimorbidity |  |  |  |
| None | – | – | – |
| 1 or 2 |  | 0.81 (0.30 – 2.21) | 0.80 (0.26 – 2.22) |
| 3 or more |  | 0.66 (0.25 – 1.72) | 0.65 (0.24 – 1.80) |
| Psychological distress |  | 0.95 (0.87 – 1.03) | 0.94 (0.87 – 1.02) |
| Social networks |  | 1.06 (0.98 – 1.14) | 1.06 (0.98 – 1.14) |
| Community attachment |  | 1.10 (0.96 – 1.26) | 1.10 (0.96 – 1.27) |
| **3. Built environment** |  |  |  |
| Park distance |  |  | 1.05 (0.88 – 1.24) |
| Kopitiam distance |  |  | 1.69 (1.00 – 2.84)* |
| ***Exercise: Every day vs. none*** |  |  |  |
| **1. Demographic** |  |  |  |
| Age |  |  |  |
| 60 – 70 | – | – | – |
| 71 – 80 | 3.04 (1.25 – 7.42)* | 3.17 (1.24 – 8.09)* | 3.05 (1.18 – 7.93)* |
| 81 – 100 | 1.54 (0.55 – 4.31) | 1.47 (0.48 – 4.51) | 1.45 (0.46 – 4.54) |
| Education |  |  |  |
| Did not attend school | – | – | – |
| Primary school | 1.47 (0.47 – 4.62) | 1.28 (0.37 – 4.46) | 1.84 (0.50 – 6.75) |
| Secondary school | 1.21 (0.37 – 4.03) | 1.14 (0.30 – 4.29) | 1.20 (0.31 – 4.64) |
| University | 5.53 (0.88 – 34.67) | 4.00 (0.58 – 27.43) | 4.52 (0.64 – 31.80) |
| Flat type |  |  |  |
| 3-room | – | – | – |
| 4/5-room | 1.73 (0.74 – 4.08) | 1.44 (0.57 – 3.63) | 0.98 (0.36 – 2.69) |
| **2. Health status** |  |  |  |
| Multimorbidity |  |  |  |
| None | – | – | – |
| 1 or 2 |  | 0.89 (0.32 – 2.49) | 0.87 (0.30 – 2.48) |
| 3 or more |  | 0.88 (0.33 – 2.32) | 0.96 (0.34 – 2.68) |
| Psychological distress |  | 0.92 (0.84 – 1.01) | 0.92 (0.84 – 1.01) |
| Social networks |  | 1.03 (0.95 – 1.10) | 1.03 (0.96 – 1.11) |
| Community attachment |  | 1.14 (0.99 – 1.31) | 1.15 (1.00 – 1.32) |
| **3. Built environment** |  |  |  |
| Park distance |  |  | 0.97 (0.81 – 1.15) |
| Kopitiam distance |  |  | 1.94 (1.14 – 3.29)* |
| McFadden’s Pseudo R^2^ | 0.052 | 0.100 | 0.118 |
| ∆ R^2^ | – | 0.048 | 0.018 |

Note: Kopitiam (traditional local coffeeshop) distance was calculated from the nearest Kopitiam. * p<.05 ** p<.01 *** p<.001.

**Table S3. Hierarchical multinomial regression for exercise among females (N=283)**

|  | Step 1 | Step 2 | Step 3 |
| --- | --- | --- | --- |
|  | Adj. OR (95% CI) | Adj. OR (95% CI) | Adj. OR (95% CI) |
| ***Exercise: Some days vs. none*** |  |  |  |
| **1. Demographic** |  |  |  |
| Age |  |  |  |
| 60 – 70 | – | – | – |
| 71 – 80 | 1.96 (0.98 – 3.93) | 1.88 (0.91 – 3.89) | 1.81 (0.87 – 3.78) |
| 81 – 100 | 0.80 (0.29 – 2.16) | 0.69 (0.24 – 1.98) | 0.62 (0.21 – 1.82) |
| Education |  |  |  |
| Did not attend school | – | – | – |
| Primary school | 1.25 (0.55 – 2.80) | 1.29 (0.55 – 3.03) | 1.29 (0.54 – 3.02) |
| Secondary school | 1.51 (0.64 – 3.53) | 1.47 (0.60 – 3.60) | 1.43 (0.59 – 3.51) |
| University | 4.26 (1.03 – 17.57)* | 4.23 (0.98 – 18.26) | 4.15 (0.97 – 17.82) |
| Flat type |  |  |  |
| 3-room | – | – | – |
| 4/5-room | 1.22 (0.63 – 2.34) | 1.26 (0.64 – 2.51) | 0.99 (0.46 – 2.15) |
| **2. Health status** |  |  |  |
| Multimorbidity |  |  |  |
| None | – | – | – |
| 1 or 2 |  | 1.43 (0.65 – 3.16) | 1.46 (0.66 – 3.24) |
| 3 or more |  | 1.97 (0.90 – 4.27) | 2.13 (0.97 – 4.68) |
| Psychological distress |  | 1.09 (0.99 – 1.19) | 1.10 (1.00 – 1.20) |
| Social networks |  | 1.11 (1.04 – 1.18)** | 1.11 (1.03 – 1.18)** |
| Community attachment |  | 1.07 (0.97 – 1.17) | 1.07 (0.97 – 1.17) |
| **3. Built environment** |  |  |  |
| Park distance |  |  | 0.91 (0.79 – 1.17) |
| Kopitiam distance |  |  | 1.00 (0.67 – 1.48) |
| ***Exercise: Every day vs. none*** |  |  |  |
| **1. Demographic** |  |  |  |
| Age |  |  |  |
| 60 – 70 | – | – | – |
| 71 – 80 | 2.31 (1.11 – 4.79)* | 2.20 (1.04 – 4.67) | 2.10 (0.98 – 4.51) |
| 81 – 100 | 1.35 (0.51 – 3.60) | 1.45 (0.52 – 4.04) | 1.33 (0.47 – 3.74) |
| Education |  |  |  |
| Did not attend school | – | – | – |
| Primary school | 0.93 (0.40 – 2.16) | 0.89 (0.37 – 2.15) | 0.90 (0.37 – 2.20) |
| Secondary school | 1.65 (0.70 – 3.91) | 1.64 (0.66 – 4.03) | 1.59 (0.64 – 3.96) |
| University | 2.72 (0.62 – 11.81) | 2.82 (0.63 – 12.62) | 2.74 (0.61 – 12.30) |
| Flat type |  |  |  |
| 3-room | – | – | – |
| 4/5-room | 2.09 (1.08 – 4.06)* | 1.87 (0.94 – 3.72) | 1.43 (0.66 – 3.10) |
| **2. Health status** |  |  |  |
| Multimorbidity |  |  |  |
| None |  | – | – |
| 1 or 2 |  | 1.47 (0.67 – 3.25) | 1.49 (0.67 – 3.31) |
| 3 or more |  | 1.35 (0.61 – 3.01) | 1.50 (0.67 – 3.38) |
| Psychological distress |  | 0.92 (0.83 – 1.01) | 0.93 (0.84 – 1.03) |
| Social networks |  | 1.08 (1.01 – 1.15)* | 1.08 (1.01 – 1.15)* |
| Community attachment |  | 1.00 (0.90 – 1.10) | 1.00 (0.91 – 1.11) |
| **3. Built environment** |  |  |  |
| Park distance |  |  | 0.88 (0.77 – 1.00) |
| Kopitiam distance |  |  | 0.95 (0.63 – 1.41) |
| McFadden’s Pseudo R^2^ | 0.032 | 0.098 | 0.103 |
| ∆ R^2^ | – | 0.066 | 0.005 |

Note: Kopitiam (traditional local coffeeshop) distance was calculated from the nearest Kopitiam. * p<.05 ** p<.01 *** p<.001.

**Figure S1. Map of Surrounding Areas of the Study Neighborhood**


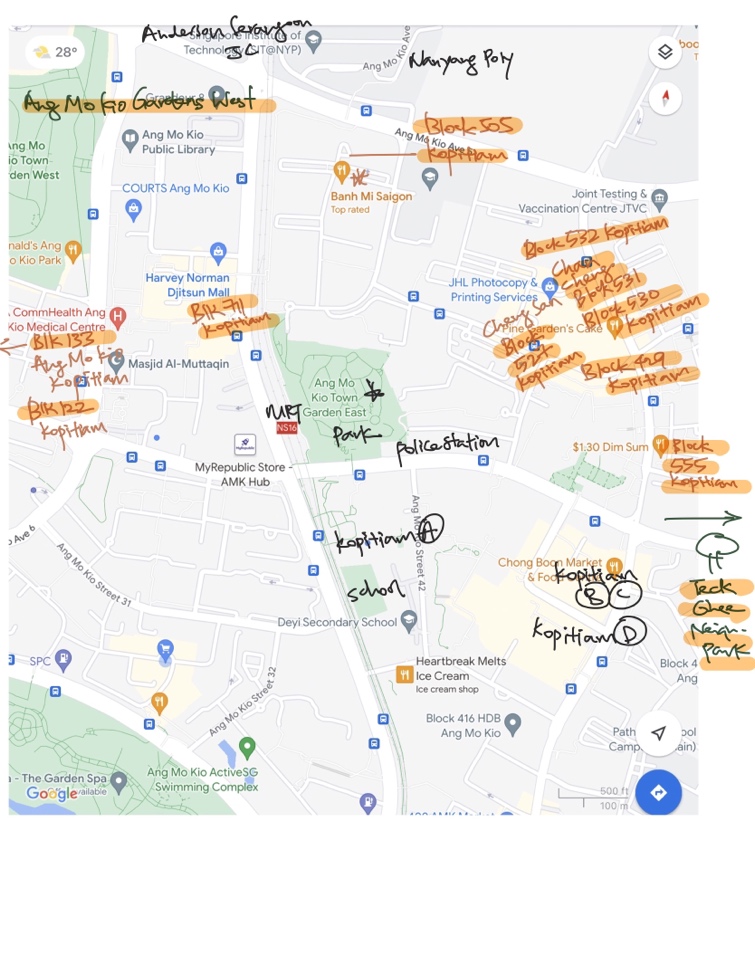


**Parks (greeneries)**

- Study neighborhood
  - Ang Mo Kio Gardens West (center)
- Other parks outside the neighborhood
  - Ang Mo Kio Gardens West (top-right)
  - Bishan Ang Mo Kio Park (bottom-right)

**Nearby Kopitiams (yellow highlighted)**

- Study neighborhood:
  - Kopitiam A, B, C, D
- Other kopitiams – yellow highlighted
